# Feasibility and Acceptability of an App-Based, Multimodal Prehabilitation Program for Women Undergoing Surgery for Gynaecological Cancer

**DOI:** 10.1177/10732748251375516

**Published:** 2025-09-16

**Authors:** Chhaya Ganda-Heath, Anke Smits, Christine Ang, Nithya Ratnavelu

**Affiliations:** 1 5994 Newcastle UniversityMedical School, Newcastle Upon Tyne, UK; 2 156807 Northern Gynaecological Oncology Centre, Queen Elizabeth Hospital, Gateshead, UK

**Keywords:** prehabilitation, app-based, gynaecological cancer, surgery, feasibility, acceptability

## Abstract

**Introduction:**

Prehabilitation aims to optimise functional capacity, nutritional status, and mental wellbeing, to minimise surgical morbidity and enhance recovery. Hospital-based prehabilitation programs may face practical barriers, such as distance, time, and cost of travel. App-based prehabilitation may therefore offer a practical solution.

**Objective:**

The aim of this study was to assess the feasibility, acceptability, and safety of introducing an app-based prehabilitation program into the standard care for women undergoing gynaecological cancer surgery.

**Methods:**

This was a feasibility study performed as part of a prehabilitation service development project for women undergoing gynaecological cancer surgery between April 2024 and May 2024. Women were approached to trial the ‘Surgery Hero’ prehabilitation app prior to surgery, which was a multimodal app-based intervention including physical exercise, nutrition, and mental wellbeing through information modules, videos, and real-time access to a health coach. Feasibility was assessed through recruitment, activation, participation, and drop-out rates, and adverse events were monitored. Acceptability was assessed through participant satisfaction scores, and semi-structured interviews with thematic analysis of facilitators and barriers to program participation.

**Results:**

Fifteen licenses were available and successfully utilised, with a recruitment rate of 58.6% and an activation rate of 88.2%. The participation rate was 93.3% and the mean engagement rate was 76.3%. No adverse events were reported. The mean overall program satisfaction score was 4.5 out of 5. Participants felt motivated to participate and were reassured by the coaching calls and information provision. However, they felt limited by the short preoperative interval and their current state of health.

**Conclusions:**

App-based prehabilitation appears to be a feasible, acceptable, and safe option for women undergoing surgery for a gynaecological cancer. However, further studies are needed to confirm these findings.

## Plain language summary

Prehabilitation is increasingly offered to women undergoing surgery to improve fitness, and it has been shown to decrease complications and improve recovery. However, participation in hospital-based prehabilitation can be difficult for patients due to requirements to travel, associated costs, and limited time before surgery. Prehabilitation via a mobile app may offer a flexible alternative to hospital-based programs.

Therefore, an app-based prehabilitation program was offered to women with gynaecological cancer undergoing surgery, through the ‘Surgery Hero’ mobile prehabilitation app. The aim was to assess whether an app-based program could be successfully introduced into routine care, and whether it was acceptable to patients. The app included informational modules and videos on physical exercise, nutrition, and mental wellbeing, and offered real-time access to a health coach for personalised advice. The feasibility of the program was measured through the number of eligible women identified, the number of women recruited that activated the app, and app usage overall. Participants were asked to provide feedback on their experience through interviews.

Most of the women who were recruited successfully activated the app, and engaged with at least one core component of the app. There were no adverse effects reported attributable to program participation. The average satisfaction score was 4.5 out of 5, and feedback was predominantly positive.

Overall, app-based prehabilitation appears to be a feasible and safe alternative to hospital-based programs that is well-received by women with gynaecological cancer who are undergoing surgery.

## Introduction

Whilst surgery is the cornerstone of treatment for most gynaecological cancers, surgery is often associated with significant risks of intraoperative and postoperative complications^1^ and long-term physical and psychosexual morbidity.^2^ In addition, this specific patient cohort is generally characterised by advanced age, high comorbidity index, frailty and obesity which further increase their risk of treatment morbidity and mortality.^3-8^

Prehabilitation is the process of preoperatively optimising a patient’s functional capacity and mental wellbeing, with the aim to improve treatment morbidity and other outcomes. Growing evidence on prehabilitation in major abdominal cancer surgery concluded that prehabilitation successfully improved functional capacity and reduced post-operative complications.^9,10^ Therefore, guidelines recommend prehabilitation for all cancer patients with exercise, nutritional and psychological components at a minimum.^11^

There are limited studies on prehabilitation in gynaecological cancer surgery.^12,13^ However, results so far have been promising, showing program feasibility with high patient participation rate,^14,15^ reduction in hospital length of stay,^15,16^ and both direct and indirect cost-savings.^17^ Women with gynaecological cancer demonstrate a willingness to participate, but unfortunately, there are practical barriers to hospital-based prehabilitation and supervised programs. These include patient accessibility in terms of travel and costs, and the short interval to surgery.^18^ These issues may be mitigated through the delivery of prehabilitation using a digital interphase.^19^ Specifically, app-based interventions have shown to be beneficial for physical fitness and psychological wellbeing in other oncological surgery settings.^20-23^

Currently, there are no studies on digital app-based, multimodal prehabilitation programs for women with gynaecological cancer. Therefore, this study aimed to evaluate the feasibility and acceptability of participating in an app-based prehabilitation program for women awaiting surgery for gynaecological cancer.

## Methods

This was a feasibility study performed as part of a prehabilitation service development project on introducing the ‘Surgery Hero’ prehabilitation digital app into the care of women with gynaecological cancer at the Northern Gynaecological Oncology Centre (NGOC), Gateshead, United Kingdom. A mixed methods design was used, incorporating quantitative and qualitive approaches to assess feasibility and acceptability. The study is reported in accordance with STROBE guidelines for cohort studies.^24^ The NGOC is a tertiary centre for gynaecological cancer care that covers the wide geographical region of the North East and Cumbria, with a dispersed population of approximately 2.6 million people and high levels of socioeconomic deprivation.^25,26^ In accordance with NHS Health Research Authority guidance,^27^ this project was exempt from formal ethical approval by a Research Ethics Committee as it was as service development project.

### Study Population and Recruitment

Women were deemed eligible for participation if they were over 18 years old, with confirmed or suspected primary gynaecological cancer undergoing surgery at the NGOC, with at least a 2-week interval until their scheduled surgery date. The exclusion criteria included: (i) any premorbid conditions, impaired mobility, or injury that contraindicates unsupervised exercise, or is exacerbated by exercise; (ii) American Society of Anaesthesiologists (ASA) grade of 4 or more; (iii) no access to a smartphone or internet connection. Caldicott Guardian Approval was provided by Gateshead Health NHS Foundation Trust to access, record and share patient identifiable information in line with local data governance principles.

Fifteen licences were purchased and made available to eligible women between the recruitment period of 22^nd^ April 2024 to 20^th^ May 2024. Consecutive women were sent participant information leaflets via post before their clinic appointment. In clinic, if eligible, they were approached to discuss participation and consented by a dedicated project member or were offered a follow-up appointment by telephone if required to consent remotely. Participants were anonymised using unique study numbers and their details were securely passed onto Surgery Hero to initiate the onboarding process. Recruitment was defined as consenting to participation in the app-based prehabilitation program.

Surgery Hero sent a text message to participants to download the Surgery Hero app from the App Store and complete the onboarding procedure. Activation was defined as successfully downloading the app and completing the in-app entry assessment. In the instance that a participant did not onboard, their license was released to be offered to another person.

### Intervention

**Figure 1.**
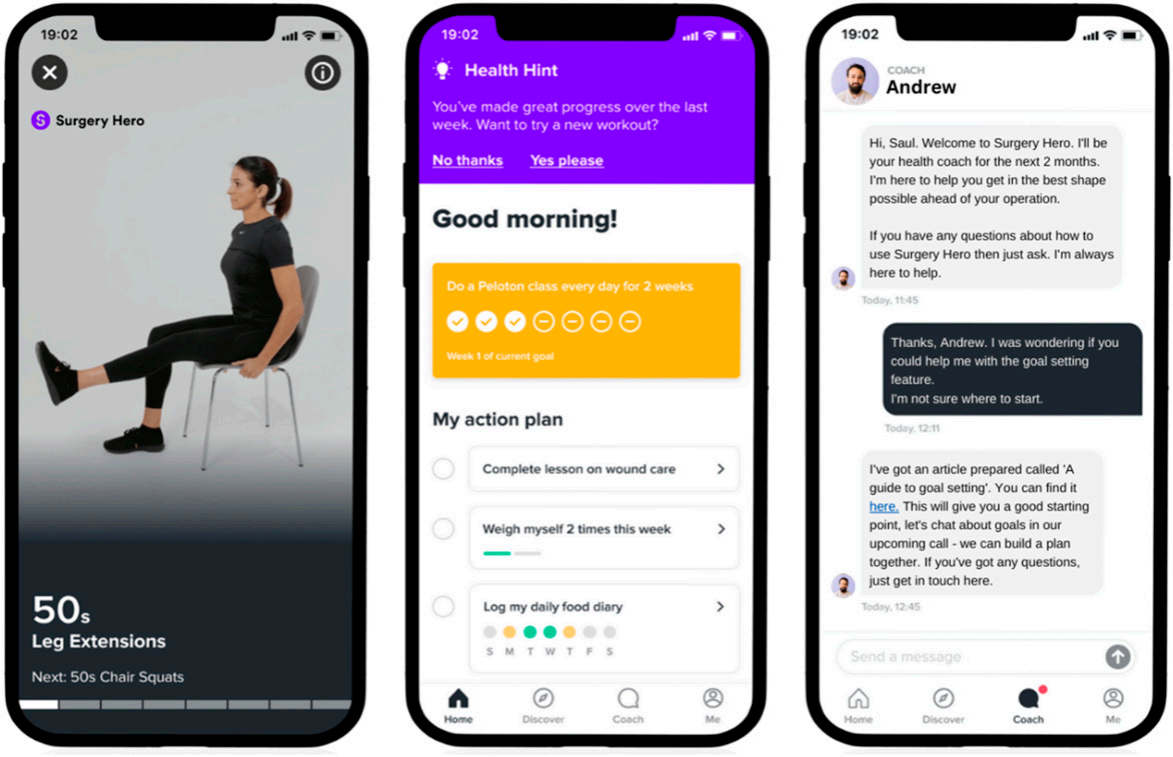
Surgery Hero App Imagery Showing Exercise Video, App Homepage With Personalised Prehabilitation Plan and In-App Health Coach Messaging

The intervention comprised 12-weeks access to the Surgery Hero mobile app and health coaching program for use before and after surgery. Surgery Hero offered a comprehensive 1:1 prehabilitation service via a hybrid human-digital approach, costing £150 plus VAT per license. The app introduced a multimodal intervention which included physical activity, nutritional and mental wellbeing. This included bite-sized educational modules on preparing for surgery; graded exercise videos to improve muscle mass and aerobic capacity; meal plans for optimal preoperative nutrition; and mindfulness techniques for anxiety reduction. A trained health coach was available to offer real-time personalised support via an in-app messaging service and calls (Figure 1). Participation was defined as usage of these core component of the app-based program. All women were offered a 30-minute ‘welcome call’ with their coach to discuss initial goals and then organised further coaching contact as desired to discuss concerns, plan preparation for surgery and monitor progress. Participants were able to track health metrics such as steps, sleep, alcohol consumption and smoking, and access group learning sessions.

Importantly, women were also able to access the app for rehabilitation after surgery to complete their 12-week program.^28^ Participants defined their personalised prehabilitation goals with guidance from their health coach and engaged with the elements that were relevant to them. Participants were asked to complete an activity self-tracker to record program participation. Surgery Hero recorded time to onboarding from text message invitation and weekly engagement with core components of the app program.

## Outcomes

The primary outcomes of the study were to assess the feasibility of introducing an app-based prehabilitation program in terms of recruitment rate, activation rate, participation rate, engagement rate, drop-out rate, and program safety. Recruitment rate was defined as the number of participants that consented out of the number of eligible women approached. Activation rate was defined as the number of participants that successfully downloaded the app and completed the in-app entry assessment. Participation rate was defined as the number of participants that used a core component of the app out of the total number of participants. Engagement rate was defined as the percentage of weeks that a participant engaged with a component of the app until their surgery date out of total weeks until surgery date. Safety was measured through frequency of adverse events attributable to program participation.

The secondary outcome was acceptability of the program to participants. Women were asked to participate in pre-intervention and post-intervention semi-structured interviews with the study team. The interview guides were co-designed with input from the gynaecological cancer clinical team and from a patient and public involvement group. The questionnaire included both scaled questions and open questions to explore acceptability in-depth. Acceptability was measured through overall program satisfaction rating and satisfaction rating for individual component, such as health coaching calls and educational modules. Interview data was used to further explore user experience and program satisfaction; to determine app usage after surgery for rehabilitation purposes; and to identify facilitators and barriers to program participation through thematic analysis.

### Data Analysis

Continuous data was presented through measures of central tendency (mean or median) and variance (IQR and range) and was assessed using t-tests and Mann-Whitney-U tests. Categorical data was presented as frequencies and proportions and was assessed using chi-squared tests and Fisher’s exact tests. Interviews were recorded, transcribed from audio files, and verified manually. Interview data was evaluated through inductive thematic analysis using NVIVO 14 software. There was no pre-defined coding frame, and only themes relevant to the aims were reported.

## Results

### Primary Outcomes

#### Recruitment, activation, and participation

**Figure 2.**
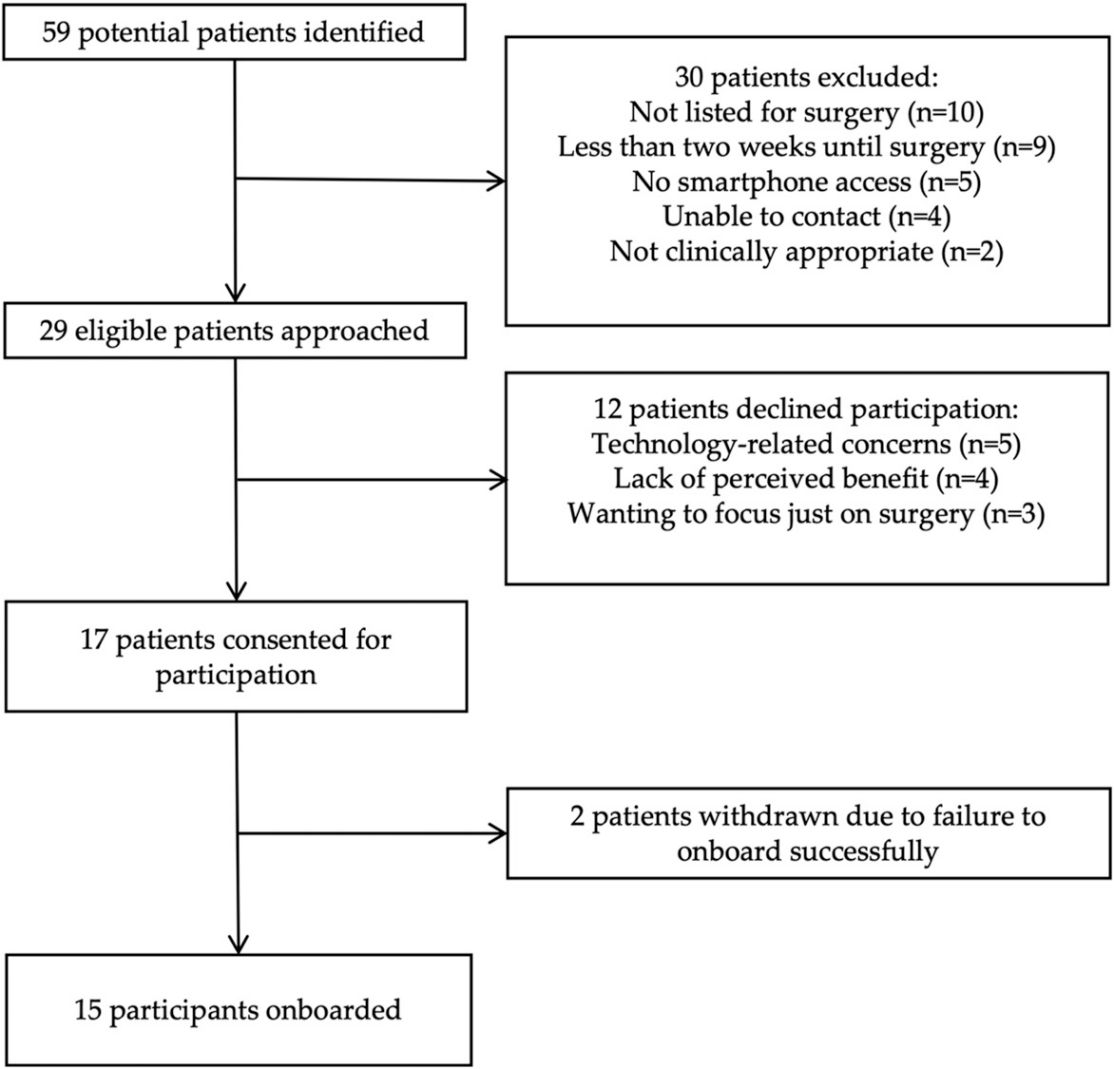
Flowchart Showing Recruitment Process

The recruitment process is outlined in Figure 2. Fifty-nine new patients were referred to the weekly regional multidisciplinary team meeting for treatment at the NGOC between 22^nd^ April 2024 and 20^th^ May 2024. Thirty women were excluded after screening for various reasons shown in Figure 2. Twenty-nine women were deemed eligible for participation and were approached in clinic, of whom twelve women declined participation. Reasons for declining participation were ‘technology-related concerns/aversion’ (n = 5), ‘lack of perceived benefit from participation’ (n = 4), and ‘wanting to just focus on surgery’ (n = 3). Seventeen women consented to participation in the app-based program, giving a recruitment rate of 58.6% (17/29). Two women did not successfully onboard, so the activation rate was 88.2% (15/17). The final cohort comprised 15 participants. Fourteen out of fifteen participants used at least one core component of the program, giving a pre-operative participation rate of 93.3%. The reason for non-participation was poor mental health. The mean engagement rate was 76.3% (range: 0%–100%).

#### Feasibility

It took participants a median 6 days (range: 3-14 days) to onboard after consenting. The median time from consent to surgery date was 20 days (range: 15-27 days), and the median time from onboarding to surgery was 14 days (range: 9-24 days). Nine women (60%) had access to the app for 14 days or more, counted from onboarding until the date of surgery. Ten women (66%) had one or more coaching calls.

There were no reported adverse events. One participant withdrew from follow-up 18 days after onboarding; this was 2 days before their surgery date. The reason for drop-out was feeling overwhelmed by their cancer journey and consistent contact from Surgery Hero. This resulted in a drop-out rate of 6.7% (1/15).

Eight women used the app after their surgery, giving a post-operative participation rate of 53.3%. The mean post-operative engagement rate was 45% (range: 20%–100%). This compared to eleven women (73%) who said they would use the app after surgery during interviews. There was no difference in baseline characteristics of women who engaged after surgery and those that did not engage after surgery. There was a significant association between greater engagement before surgery and usage after surgery. *(P = 0.005)*.

#### Participant characteristics

Baseline participant demographic and clinical characteristic are shown in Table 1. The median age was 62 years (IQR: 56 – 69 years; range: 42 – 83 years). Twelve women were diagnosed with gynaecological cancer, and 3 women were diagnosed with benign pelvic mass. The most prevalent diagnosis was ovarian cancer (n = 8; 53%). The median BMI was 27.6 kg/m^2^ (range: 19.8 -50.3 kg/m2). Most participants (53%) had 3 or more comorbidities. Ten participants (67%) had a Rockwood Frailty Score between 1 (very well) and 3 (managing well). The median distance to travel to the NGOC was 22 miles (range: 2 miles - 66 miles).

**Table 1.** Baseline Participant Clinical and Demographic Characteristics

Baseline patient characteristics	Participants n = 15
Age (years), median (IQR; range)	62 (56-69; 42-83)
Baseline weight (kg), median (IQR; range)	73 (67-95; 51-121)
Baseline BMI (kg/m2), n (%)	
<18.5	0 (0)
18.5-24.9	4 (27)
25.0-29.9	5 (33)
≥30	6 (40)
Diagnosis, n (%)	
Ovarian cancer	8 (53)
Uterine cancer	1 (7)
Cervical cancer	0 (0)
Vaginal cancer	1 (7)
Vulval cancer	2 (13)
Pelvic mass	3 (20)
Time to surgery (days), median (IQR; range)	20 (16-23; 15-27)
Comorbidities, n (%)	
0	2 (13)
1	0 (0)
2	5 (33)
≥3	8 (53)
Regular medications, n (%)	
0	3 (20)
1	2 (13)
2	3 (20)
≥3	7 (47)
Rockwood frailty score, n (%)	
1-3	10 (67)
4-6	5 (33)
7-9	0 (0)
Smoking status, n (%)	
Never smoked	8 (53)
Previous smoker	5 (33)
Current smoker	2 (13)
Alcohol consumption, n (%)	
Never drinks	5 (33)
Social drinking only	6 (40)
Drinks regularly	4 (27)
Occupational status, n (%)	
Employed	6 (40)
Retired	9 (60)
Index of multiple deprivation decile, n (%)	
1-3	5 (33)
4-7	7 (47)
8-10	3 (20)
Distance to NGOC from postcode [miles], median (IQR; range)	22 (9-46; 2-66)

## Secondary Outcomes

### Acceptability and Satisfaction

The app-based program demonstrated high patient acceptability and satisfaction rates, shown in Table 2. The mean overall program satisfaction score was 4.5 out of 5 (range: 3.5 – 5). One participant had trouble with the onboarding process, but all other participants reported that the onboarding process was simple. There was no difference in mean overall satisfaction between those who engaged with their coach, and those who did not. The reasons for not engaging with a health coach were participant choice (n = 3), Surgery Hero-related issues such as delayed onboarding (n = 1) and lack of available appointments (n = 1). The mean satisfaction with coaching calls was 4.5 out of 5 (range: 4 – 5).

**Table 2.** Mean Participant Satisfaction Scores

Mean participant satisfaction scores	Result
Mean overall program satisfaction [score out of 5], n (range)	4.5 (3.5 – 5)
Mean ease of onboarding [score out of 5], n (range)	4.7 (1 – 5)
Mean satisfaction with coaching calls [score out of 5], n (range)	4.5 (4 – 5)
Mean satisfaction with information and activities within the app [score out of 5), n (range)	4.6 (3.5 – 5)
Mean satisfaction with prehabilitation knowledge before participation [score out of 5], n (range)	4.3 (3 – 5)
Mean satisfaction with prehabilitation knowledge after participation [score out of 5], n (range	4.5 (4 – 5)
Number of technical issues reported	1

### Facilitators and Barriers

**Figure 3.**
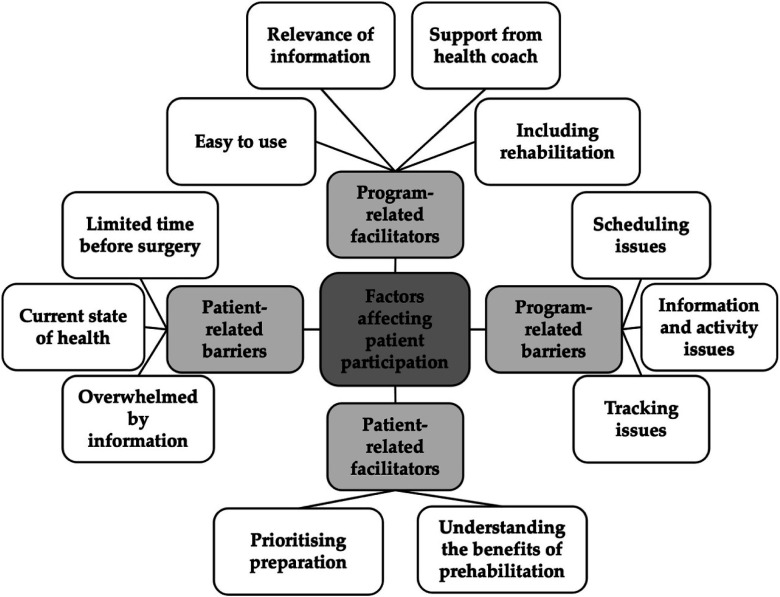
Thematic Map Demonstrating Facilitators and Barriers to Program Participation and Satisfaction, Derived From Semi-structured Interviews

Facilitators and barriers to patient participation were organised into 4 main themes, shown in Figure 3: patient-related facilitators; program-related facilitators; patient-related barriers and program-related-barriers. Further details can be found in Appendix 2.

Participants recognised the importance of prehabilitation and its benefits, and this encouraged prioritisation of participation and motivated positive changes to health behaviours. Health coaches improved motivation to participate and provided reassurance as a consistent point of contact. All women who engaged with their health coach derived benefit from the interaction, contributing to program satisfaction. The inclusion of the rehabilitation component was a key motivator for participation as it offered continuous support throughout the whole journey. The app information modules covered important elements of preparation in an easily accessible manner that could be personalised based on the woman’s goals and discussions with their health coach. Participants felt the information modules were the most beneficial element of the program.

Some women struggled with participation due to physical symptoms of cancer, side effects of treatment and poor mental health; these factors physically limited participation and reduced motivation to use the app daily. Participants perceived that the short time to surgery was the main limitation to participation and satisfaction, especially as they often had other priorities and key life events during the preoperative period. There were some technological issues with the app that limited satisfaction and user experience, such as slow loading times and difficulties with tracking metrics. Additionally, there were organisational issues that impacted satisfaction and perceived benefit, namely scheduling health coaching calls and missing group sessions due to late notice.

## Discussion

This is the first reported study of an app-based, multimodal prehabilitation program for women with a (suspected) gynaecological cancer, irrespective of cancer type or surgical approach. This study shows that app-based prehabilitation for gynaecological cancer surgery is feasible and safe, with recruitment and activation rates of 58.6% and 88.2% respectively. Overall satisfaction was high and participant feedback was predominantly positive, indicating acceptability within this patient cohort.

Previous studies have demonstrated feasibility of multimodal prehabilitation programs for gynaecological cancer patients with recruitment rates of 73% and 90% for community and hospital-based programs respectively.^14,15^ The main barriers identified in this study were analogous with previous literature, such as lack of perceived benefit and the perception of participation as an additional burden.^14,18^ Studies evaluating app-based prehabilitation in other oncological surgical specialities are also scarce, and few comment on feasibility in terms of recruitment and activation rates. A study on app-based prehabilitation in oesophago-gastric cancer patients reported a recruitment rate of 75%, however 40% of these patients did not possess the digital literacy to use the app, limiting direct comparison.^20^ Five women were excluded due to lack of smartphone, and 5 women declined due to technology-related concerns such as lack of digital confidence. A previous survey on app-usage in cancer care that reported 43.5% of their participants refused to send medical data via an app due to poor digital skills and concerns about data safety.^29^ Provision of smartphones on loan to those without would promote access to the intervention and minimise exclusion.^30^ Additionally, offering 1:1 training with a digital care navigator or group digital skills sessions may promote digital inclusion to improve recruitment.^20,31^

Understanding the benefit of tailored prehabilitation and encouragement to participate by their clinician were identified as a key motivator for participation in this study and others.^18,32-34^ Further education of clinicians is needed on initiating discussions on the benefits of personalised prehabilitation as part of standard care in this population, and their role in motivating patient recruitment and participation. Education is an essential part of recruitment, especially as many patients were unfamiliar with the concept of prehabilitation and were therefore less likely to prioritise it before surgery.^33^ In addition, the suggested use of social media to improve education on prehabilitation and allowing self-referral to the intervention may also improve recruitment, which was shown to be feasible in another study.^21^

Previous studies support this study’s findings that both patients and healthcare professionals feel the short preoperative period within the cancer pathway is the primary barrier to prehabilitation.^18,32^ The onboarding process took an average of 6 days and only 60% of participants achieved the minimum 2 weeks of prehabilitation suggested in the guidelines.^11^ Whilst this may be acceptable for other surgical prehabilitation, it drastically reduces the time for prehabilitation within the expedited cancer surgery pathway in the National Health Service, where cancer treatment must be completed within a maximum of 62 days of presentation and within 31 days from decision to treat date.^35^ Despite agreement that prehabilitation is valuable, neither patients nor healthcare professionals appear willing to delay surgery due to the fear of poorer outcomes.^18,32^ Therefore, the onboarding process requires streamlining, possibly through additional study coordinators in clinic and fast-track onboarding pathways.^36^ Given the remote nature of the program, initiation of prehabilitation upon referral from primary care could be possible; further research on this is warranted to assess feasibility.

The mean overall satisfaction with the program was high, as seen in other app-based programs.^37,38^ Addressing feedback on technical and logistical challenges could further enhance the user experience and improve engagement with the program. Interestingly, this study found that app-based prehabilitation was acceptable to participants across all age groups. The cohort’s median age was higher compared to other app-based studies,^22,29,37^ suggesting that app-based program may be feasible even for the ageing population - especially given contemporary reports that 67% of adults over 65 own a smartphone.^39^

There is an important argument to be made for app-based prehabilitation because it provides an alternative method to deliver personalised programs from home without compromising program adherence, which is notably lower in unsupervised programs.^40^ Studies within gynaecological and other major abdominal cancer surgery show that patients, especially women, were more likely to participate in home-based prehabilitation programs that fit into daily life, and app-based programs may offer this flexibility.^18,32,33^ App-based programs reduce travel, transport costs and the time spent in hospital; this is particularly important in this cohort as patients already have a high appointment burden and receive their care in specialised tertiary centres across a wide geographical area.^18,33,41,42^ Moreover, app-based prehabilitation does not require additional infrastructure or staffing, potentially offering substantial direct and indirect cost-savings compared to hospital-based programs. However, cost analysis was beyond the scope of this study, and heterogeneity in program design and delivery limited meaningful cost comparison.^38,43^

In addition, whilst rehabilitation was beyond the scope of this study, the inclusion of prehabilitation and rehabilitation offered within this program was a key motivator for participation in some women. Notably greater participation in prehabilitation was associated with higher app usage after surgery. This suggests that improving participation with prehabilitation may help improve engagement with rehabilitation and facilitate sustained long-term behavioural change.

This is the first report of an app-based prehabilitation program for women undergoing surgery for a (suspected) gynaecological cancer. The Surgery Hero app has been implemented across the UK in various specialities such as orthopaedics and colorectal surgery,^28,38^ but this study is the first to pilot the app in gynaecological cancer. A key strength of the study is the inclusion of all gynaecological cancers, including vulval and vaginal cancer, where previous studies have focussed on ovarian or endometrial cancer only.^12,15,16^ This study benefits from multimodal intervention where most app-based studies have been unimodal, primarily focusing on psychological wellbeing alone.^21,22^

It is recognised that this was a small, single-institution study of a service development project at a tertiary centre serving a wide geographical area with a socio-economically diverse population. Therefore, certain caution is warranted when extrapolating these findings to other centres. The primary limitation of the small sample size limits further meaningful statistical analysis, validity, and generalisability. Additionally, there is a potential risk of selection bias, as women who were willing to participate may differ from the wider target population in terms of motivation and digital literacy, further affecting generalisability. Participant feedback was collected retrospectively which may introduce potential recall bias around engagement and satisfaction. Whilst Surgery Hero provided summary data on overall weekly engagement, more granular data on daily participation and individual program components was not available. However, this was partially mitigated by incorporating qualitative data, which offered insights on participation behaviours and barriers.

### Future Work

Future studies should aim to evaluate the feasibility and acceptability of app-based prehabilitation in a larger sample to improve the generalisability of these findings and enable meaningful subgroup analysis on variables such as age, cancer type and stage, and comorbidity index. The facilitators and barriers to participation and satisfaction identified should be considered in future study design to optimise engagement and user experience. Collecting comprehensive data on daily app usage, engagement with individual components and more detailed satisfaction metrics would offer deeper insights into participation behaviours. Further research should also assess the impact of app-based prehabilitation on surgical and oncological outcomes such as complication rates and length of hospital stay, and to compare its efficacy to hospital-based programs. Incorporating a validated tool to identify women most likely to engage with the intervention could improve allocative efficiency and cost-effectiveness. Finally, long-term follow up after surgery is warranted to evaluate the sustained impact of prehabilitation on health behaviours.

## Conclusions

This was the first study to assess the implementation of a multimodal, digital app-based prehabilitation program for women undergoing gynaecological cancer surgery. It demonstrates that app-based prehabilitation may be feasible and acceptable to women with high satisfaction rates and positive feedback on information modules and health coaching calls. App-based prehabilitation could represent a practical low-cost alternative to hospital based-programs. However, larger studies are needed to confirm this. In addition, key facilitators and barriers to participation identified in this study, such as patient state of health and patient preference for accessible programs, should be used to further guide the service design of app-based prehabilitation programs.

## Data Availability

The raw data supporting the conclusions of this article will be made available by the authors upon reasonable request.*

## Appendix 1 – STROBE Checklist^24^

STROBE Statement—Checklist of items that should be included in reports of ***cohort studies***

**Table:**

	Item No	Recommendation	Page No
**Title and abstract**	1	(*a*) Indicate the study’s design with a commonly used term in the title or the abstract	1
		(*b*) Provide in the abstract an informative and balanced summary of what was done and what was found	1
**Introduction**			
Background/rationale	2	Explain the scientific background and rationale for the investigation being reported	2
Objectives	3	State specific objectives, including any prespecified hypotheses	2
**Methods**			
Study design	4	Present key elements of study design early in the paper	2
Setting	5	Describe the setting, locations, and relevant dates, including periods of recruitment, exposure, follow-up, and data collection	2 - 3
Participants	6	(*a*) Give the eligibility criteria, and the sources and methods of selection of participants. Describe methods of follow-up	2
		(*b*) For matched studies, give matching criteria and number of exposed and unexposed	
Variables	7	Clearly define all outcomes, exposures, predictors, potential confounders, and effect modifiers. Give diagnostic criteria, if applicable	3 – 4
Data sources/ measurement	8*	For each variable of interest, give sources of data and details of methods of assessment (measurement). Describe comparability of assessment methods if there is more than one group	*3 – 4*
Bias	9	Describe any efforts to address potential sources of bias	4
Study size	10	Explain how the study size was arrived at	3
Quantitative variables	11	Explain how quantitative variables were handled in the analyses. If applicable, describe which groupings were chosen and why	4
Statistical methods	12	(*a*) Describe all statistical methods, including those used to control for confounding	4
		(*b*) Describe any methods used to examine subgroups and interactions	
		(*c*) Explain how missing data were addressed	
		(*d*) If applicable, explain how loss to follow-up was addressed	
		(*e*) Describe any sensitivity analyses	
**Results**			
Participants	13*	(a) Report numbers of individuals at each stage of study—eg numbers potentially eligible, examined for eligibility, confirmed eligible, included in the study, completing follow-up, and analysed	4 - 5
		(b) Give reasons for non-participation at each stage	4- 5
		(c) Consider use of a flow diagram	5
Descriptive data	14*	(a) Give characteristics of study participants (eg demographic, clinical, social) and information on exposures and potential confounders	6 - 7
		(b) Indicate number of participants with missing data for each variable of interest	
		(c) Summarise follow-up time (eg, average and total amount)	
Outcome data	15*	Report numbers of outcome events or summary measures over time	4 - 9
Main results	16	(*a*) Give unadjusted estimates and, if applicable, confounder-adjusted estimates and their precision (eg, 95% confidence interval). Make clear which confounders were adjusted for and why they were included	4 - 9
		(*b*) Report category boundaries when continuous variables were categorized	
		(*c*) If relevant, consider translating estimates of relative risk into absolute risk for a meaningful time period	
Other analyses	17	Report other analyses done—eg analyses of subgroups and interactions, and sensitivity analyses	
**Discussion**			
Key results	18	Summarise key results with reference to study objectives	9
Limitations	19	Discuss limitations of the study, taking into account sources of potential bias or imprecision. Discuss both direction and magnitude of any potential bias	11
Interpretation	20	Give a cautious overall interpretation of results considering objectives, limitations, multiplicity of analyses, results from similar studies, and other relevant evidence	9 - 11
Generalisability	21	Discuss the generalisability (external validity) of the study results	9 - 11
**Other information**			
Funding	22	Give the source of funding and the role of the funders for the present study and, if applicable, for the original study on which the present article is based	12

^a^Give information separately for exposed and unexposed groups.

**Note:** An Explanation and Elaboration article discusses each checklist item and gives methodological background and published examples of transparent reporting. The STROBE checklist is best used in conjunction with this article (freely available on the Web sites of PLoS Medicine at https://www.plosmedicine.org/, Annals of Internal Medicine at https://www.annals.org/, and Epidemiology at https://www.epidem.com/). Information on the STROBE Initiative is available at https://www.strobe-statement.org.

## Appendix 2 Extended Thematic Analysis

‘Patient-related facilitators’ described how participants were motivated to engage in prehabilitation when they understood its benefits and importance, and when they chose to prioritise preparation for surgery. Improving knowledge on the impact of prehabilitation encouraged patients to make more conscious health decisions, driven by the belief they could improve surgical outcomes through actions within their control. The cancer diagnosis itself motivated patients to prioritise their health and set achievable goals in the limited time before surgery. The level of engagement was indicative of participants’ personal sense of heath responsibility: those who prioritised prehabilitation were often individuals who practised health-seeking behaviours and took ownership over their health through self-management and working collaboratively with healthcare professionals. Participants also reported feeling more prepared and informed compared to previous experiences of surgery, which highlighted the importance of participating in prehabilitation.

‘Program-related facilitators’ described how desirable elements of the program facilitated participation and sustained motivation throughout its duration. All participants reported the app was easy to set up. Most participants felt the program addressed all relevant aspects of preparation for surgery, and even those already confident with healthy living reported learning something new. The information modules were presented in digestible, easy-to-follow segments and were considered the most beneficial part of the program (n = 12). Setting goals within the app helped personalise the experience, which was further enhanced by access to a personal health coach. Indeed, health coaches were identified as a significant motivator for continued participation and overall program satisfaction, providing guidance and reassurance while also holding participants accountable to improve their wellbeing (n = 9). Additionally, participants appreciated the inclusion of a rehabilitation component to the program because it offered continuity throughout the whole journey and made them felt supported by having the same health coach before and after surgery.

Patient-related barriers described factors that limited motivation to engage with the program and factors that physically limited participation. Physical symptoms of cancer and side effects of systemic treatment such as fatigue, weakness, and reduced intake sometimes limited participation in the exercise component of the program. These physical symptoms in addition to poor mental health also reduced motivation to use the app daily. The cancer diagnosis itself and the volume of information received from various healthcare professionals was sometimes overwhelming and participation in the program felt like an additional burden. The short time to surgery was reported as the core issue, especially as participants often had other priorities and key life events during the preoperative period. Participants felt they did not have enough time to optimise their health and perceived greater benefit from longer participation.

‘Program-related barriers’ described technological and logistical challenges that limited both engagement and program satisfaction. Slow reload times between information modules and difficulty inputting data retrospectively in the activity tracker hindered app usability and user experience. Some participants noted the absence of music in the exercise videos reduced their motivation to engage with this component. Attendance to group coaching calls was restricted by short notice and scheduling conflicts in the busy period. Additionally, 2 participants experienced delays in scheduling their coaching calls which impacted satisfaction – particularly given the short time to surgery and the key role health coaching played in the program.
